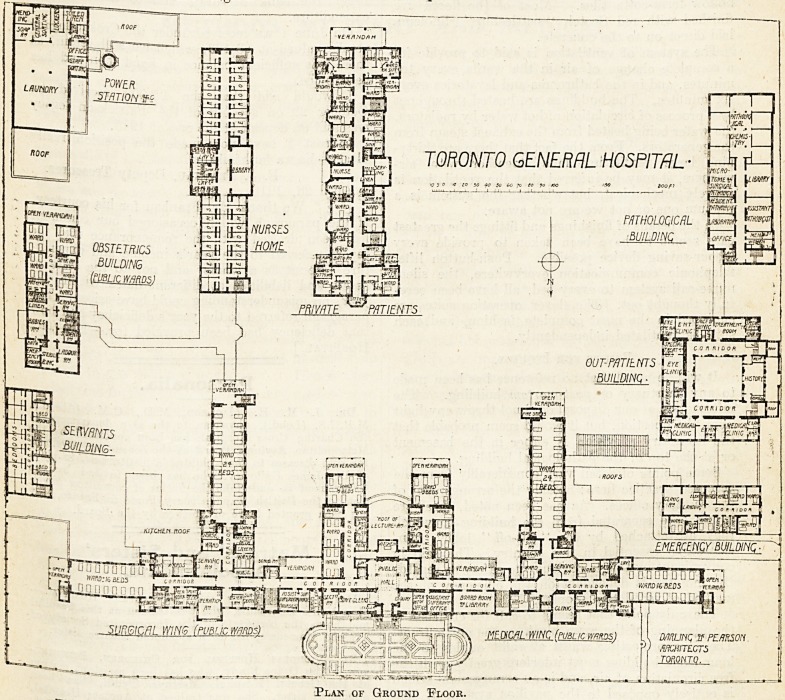# Toronto General Hospital: Accommodation 700 Patients

**Published:** 1916-05-06

**Authors:** 


### HOSPITAL ARCHITECTURE AND CONSTRUCTION.

### TORONTO GENERAL HOSPITAL.

### ACCOMMODATION 700 PATIENTS.

The site on which this hospital stands is bounded on all sides by streets, and is 9 acres in extent. The total number of patients is 700. The area of site per patient is therefore 560 ft. The main block faces College Street. In addition to this there are eight other blocks, three of which—the private patients' block, the servants' block, and the power station—are detached, the other blocks being connected with the main building by corridors at the basement level.

#### The Basement Plan.

In the basement of the main building, the central portion contains a large lecture theatre for the use of University students, and also for giving clinical lectures to practitioners throughout the city; waiting and cloak rooms for male and female students, linen-room, store-rooms, sterilising work-room, registrar's room, and housekeeper's room.

The east wing contains the kitchen offices, general stores, dining-room for male and female servants, and quarters for men-servants.

The west wing at the south end contains the admission offices and examination-room for in-patients. Here are situated six small wards for the observation of suspicious or doubtful cases, with a small kitchen and sanitary offices. Adjoining the examination-room is a large bathroom, and close by are two large stores for patients' clothes. In the front portion will be found the electrical department, consisting of $x$-ray room, photographic-room, room for the taking of histories, and one for electrical treatment. On the opposite side of the corridor is the hydro-therapeutic department. This department is fitted up with baths, in which patients can be immersed for days at a time in running water kept at a constant temperature, for the treatment of severe burns, and acute and alcoholic delirium. Baths for Nauheim treatment, electric, hot-air, and vapour cabinets, shower, needle, and spray baths are all provided for in this department.

Access to the $x$-ray and the hydrc-therapeutic department is provided direct from College Street, as well as from the hospital itself. The extreme end of the front block is occupied by a large recreation-room for nurses.

#### The Ground-Floor Plan.

On the ground floor of this main block will be found the general entrance to the hospital, and in the centre of the front or north side is the board-room and secretary's and other administrative offices.

The two spurs projecting to the south in the centre contain together thirty-five beds for semi-public patients of all classes. At the extreme south end of each of the four projecting ward blocks is an open verandah. On the south side of the main corridor there are two large verandahs, and at the ends of the sixteen-bed wards are similar verandahs. Escape staircases are planned at the extreme end of each ward block.

The first floor of the central part of the block contains a public ward for forty-four beds for treatment of eye, ear, nose, and throat, and has two operating-rooms to one suite.

The third floor contains forty beds for the treatment of gynæcological cases.

On the top floor is accommodation for twenty-seven doctors.

#### The Wards and their Adjuncts.

The east wing contains surgical wards, and the west wing the medical wards. Each wing contains two large wards, one running north and south, containing twenty-four beds, the other placed east and west, and containing sixteen beds. On the surgical side there are, in addition, one ward of three beds, two wards of one bed, and one ward of two beds.

A sink-room, medicine-room, and bathroom to each large ward, with sanitary offices and operation suite, consisting of operation-room, surgeons' room, ether-room, preparation, splint, and sterilising room. No attempt is made to cut off the sanitary offices, ventilation being apparently provided for by a large shaft. The w.c. provided for the nurses has not even any direct light.

On the medical side the arrangements are much the same, except that there are four wards for three beds and only one for one bed, and that instead of an operation theatre there is a room for clinical teaching.

The surgical wing has two storeys above the ground floor each similarly arranged, and the same applies to the medical wing. In both these wings the rooms are arranged for the use of convalescent patients, the lift in each case running up to the roof level.

#### Other Buildings.

Turning now to the buildings on University Avenue. These are three in number—the Shields emergency building, the out-patients' department, and the pathological building. Between the emergency building and the out-patients' department is the main entrance for out-patients, which is connected by a corridor to both buildings.

There is a large yard which is partially covered, into which the ambulances drive.

The emergency building is two floors high, and contains on the ground floor three wards for accidents, preparation, anæsthetic and operation rooms, examination-room, ward kitchen, linen store, and sink-room. On the upper floor are eight wards, public, private, and semi-private, with a large room for clinical teaching.

The out-patients' department also is two storeys high, and is approached direct from University

Avenue. A lobby, at each end of which is a small waiting-room, gives access to the general waiting-room, on three sides of which the various departments are arranged.

On the north side is the gynæcological room, with two examination-rooms. Next to this is the nurses' room, with sterilising apparatus. On the other side of the passage connecting this to the out-patients' department is a large cloak-room for doctors, with lavatories adjoining.

The whole of the space on the east and part of the south side is taken up by the surgical department, which comprises the general operation and recovery rooms, a small secondary waiting-room, and a room for genito-urinary work. On the south-west corner is the dispensary, and adjoining that is a corridor leading to the pathological block. The upper floor contains the medical clinic and the eye and ear department.

The site is bounded on the north by College Street, on the south by Christopher Street, on the east by Elizabeth Street, and on the west by University Avenue.

The pathological block contains on the ground floor a large laboratory for bacteriology, eight separate rooms for pathological histology, the media-room, research-room, and lecture-room. On the upper floor are rooms for pathological chemistry, a room for surgical pathology, microtome, library, and rooms for the assistant and resident pathologists, some laboratories and offices.

### A Building for 150 Private Patients.

The long building running north and south and with one end on Christopher Street is the private patients' building. This building is five storeys high, including basement, and has accommodation for 150 patients. There are wards varying in size from four beds to one.

The building is self-contained and has its own kitchen offices, also a demonstration kitchen for teaching nurses. The approach to the building is from Christopher Street, and the entrance is in the middle of the block. Every room is supplied with a fixed washing-basin and, as a rule, one bathroom is provided for each two rooms.

There is direct telephonic communication between each room and the offices.

### The Nurses' Home.

Facing the private patients' block is the nurses' home, which communicates by a corridor on the basement level with the surgical block.

Plan of Ground Floor.

This building is five storeys high with basement. On the ground floor is a dining-room with pantry adjoining and the lecture-room. In the centre of the block is a large reception-room, facing which is the main entrance with offices and library on either side. The remainder of the block is given up to bedrooms for nurses, with a bedroom and sitting-room each for the head nurse and assistant head nurse. The upper floors are occupied by bedrooms, lavatories, and bathrooms, and on the ground floor is a large library. A wide verandah on the east side overlooks the nurses' tennis court.

The small detached building on the Elizabeth Street front and close to the main building is the servants' home. This building would appear to be as high as the nurses' home, and apparently the servants are housed two in a room. There would appear to be only two sitting-rooms for the whole building, which would seem to be somewhat inadequate.

To the south of the servants' building is the obstetrics building. This building is three storeys in height with basement, is entered from Elizabeth Street, and is also connected with the main block and the nurses' home by a corridor at the ground-floor level.

A small out-patients' department is located in the basement and is entered by a separate staircase at the south end. The whole block provides accommodation for fifty patients, with the usual labour-rooms, sterilising and babies' rooms on each floor. At the south-east corner of the site is the power station, which includes boiler-house, engine-room, refrigerating plant, workshops, and laundry.

Details of Construction and Equipment.

The buildings are throughout constructed of fire-resisting materials, the outer walls being of solid brick, with a sparing use of stone dressings; the floors are of concrete, and the partition walls of hollow terra-cotta tiles. Most of the floors are finished with red Scotch "battleship" linoleum laid direct on to the concrete.

The system of ventilation is said to provide for a complete change of air in the wards every ten minutes, and in the bathrooms and lavatories every six minutes. The buildings are heated throughout by a process of circulation of hot water by radiators, the water being heated from the exhaust steam from the generators. From the fact that there are thirty-nine large ventilating fans, together with air-washers, it may be inferred that the ventilation is entirely mechanical, but whether the system is a Plenum one or not we are not aware.

In the details of finishings and fittings the greatest care seems to have been taken to provide every labour-saving device possible. Push-button lifts, telephonic communication everywhere, the silent nurse-call system to every bed, all have been carefully thought out. The eleven operation suites are equipped in the most complete fashion, and each suite is ventilated independently.

Points for Inquiry.

It will be noted that no reference has been made to any mortuary or *post-mortem* building. The information at our disposal does not throw any light upon the question, but it would seem probable that the necessary provision is either in the basement or at the top of the pathological building.

Some points of detail vary considerably from the accepted practice here; notably the arrangement of the sanitary offices. As has been noted, these are in no case separated from the building to which they are attached by the "cut-off" lobby, considered so essential in this country. The gain in convenience by this is obvious; its safety as regards the inmates is dependent on the efficiency of the method of ventilation adopted.

The only point that seems to call for criticism is the planning of the main block on the north front. The four projecting wings at right angles to the long front building must interfere greatly with the free circulation of air, and is an arrangement which is entirely opposed to the pavilion system of hospital planning. On such a magnificent site as this it surely would have been possible to have planned the main building more on the lines of modern practice.

The architects for the building are Messrs. Darling and Pearson, of Toronto.

We regret that owing to pressure on our space we are obliged to hold over the account and plans of the Calcutta Hospital for Tropical Diseases.

## Figures and Tables

**Figure f1:**
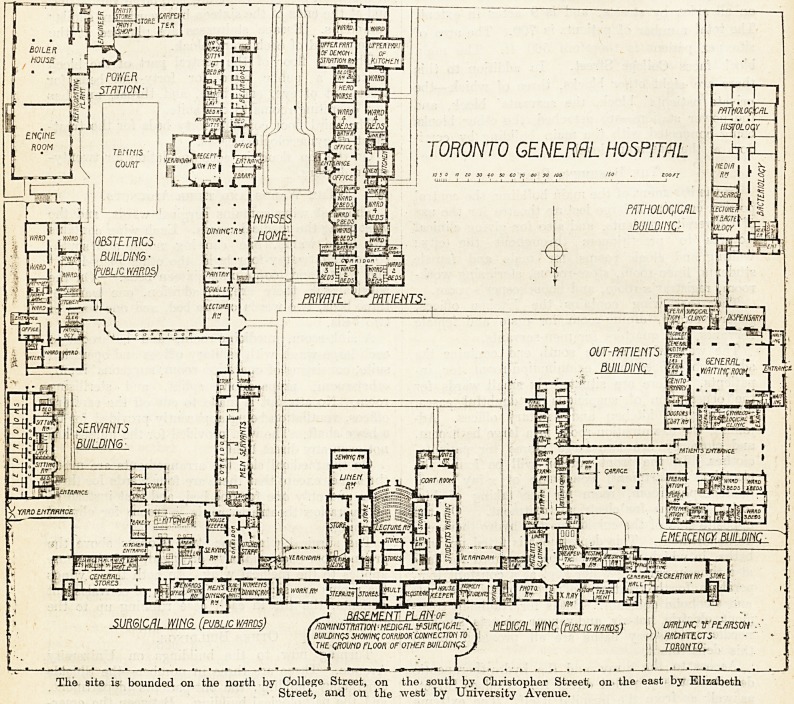


**Figure f2:**